# Magnetic iron-oxide nanoparticles in the brain connected to alcohol-associated liver disease

**DOI:** 10.1038/s41598-025-09756-8

**Published:** 2025-07-08

**Authors:** Leon Kaub, Stefan Milz, Nirav Barapatre, Andreas Büttner, Bernhard Michalke, Christoph Schmitz, Stuart A. Gilder

**Affiliations:** 1 Department of Earth and Environmental Sciences, LMU Munich, Theresienstr. 41, Munich, 80333 Germany; 2 Department of Anatomy II, Faculty of Medicine, LMU Munich, Pettenkoferstr. 11, Munich, 80336 Germany; 3 Institute of Forensic Medicine, Rostock University Medical Center, St.-Georg-Str. 108, Rostock, 18055 Germany; 4 Research Unit Analytical BioGeoChemistry, Helmholtz Center Munich, Ingolstaedter Landstr. 1, Neuherberg, 85764 Germany

**Keywords:** Brain magnetite, Brain iron, Iron overload, LA-ICP-MS, Alcohol use disorder, Alcoholic liver disease, Iron, Medical toxicology, Neuroscience, Magnetic properties and materials

## Abstract

Magnetic iron-oxide nanoparticles in the form of magnetite (Fe_3_O_4_) are present in the human brain. They have been hypothesized to biomineralize in situ, as a result of dysfunctional iron homeostasis related to Alzheimer’s disease, or to enter the brain as airborne pollution particles. Regardless of their origin, magnetic iron-oxides pose a potential hazard to human health due to their high redox activity and surface charge. Here we report measurements on four post-mortem human brainstems, with one brainstem showing approximately 100 times higher magnetite concentrations than the other cases. This brainstem came from a subject with alcohol-associated liver disease (ALD) that manifested in liver cirrhosis and massive hepatic iron overload. Laser ablation – inductively coupled plasma – mass spectrometry showed the highest levels of trace metals (iron, copper and manganese) in the ALD brainstem. It is well established that a dysfunctional liver can result in the accumulation of trace metals in the brain. Our data indicate a similar pathway for magnetite particles, yet liver pathology has not been linked to magnetite occurrence in the brain so far. It may prove to be a crucial factor in understanding the high variation of magnetite concentrations found in human brains.

**Supplementary Information:**

The online version contains supplementary material available at 10.1038/s41598-025-09756-8.

## Introduction

Iron in the brain serves important roles for many biochemical processes, including oxygen transport, oxidative phosphorylation, myelination and the synthesis of neurotransmitters^1^. Most of the iron in the brain is bound to either hemoglobin, ferritin, hemosiderin or transferrin^2^. In these forms, iron does not carry a magnetic remanence^3,4^. Nevertheless, multiple studies using independent methods have confirmed the presence of ferrimagnetic iron-oxide nanoparticles in the form of magnetite (Fe_3_O_4_) in the brain^5–8^. Their origin, potential physiological function and pathological risk are topics of ongoing research. The detection of well-shaped, euhedral crystals that resembled magnetite particles formed in magnetotactic bacteria led to the hypothesis that endogenously formed magnetite may provide a base for magnetoreception in humans^8,9^. In contrast, exogenous, pollution-based magnetite with high-temperature, combustion-derived crystal morphologies co-associated with non-physiological metals was found in the brain^5,7,10^. These magnetite particles were proposed to enter the brain through the olfactory nerve pathway^7^. Magnetite particles have also been detected in cores of amyloid-β plaques, which are the main form of senile plaques in Alzheimer’s disease (AD), and were therefore associated with dysfunctional iron homeostasis^11,12^. However, two recent studies showed no significant difference in magnetite concentrations between AD and non-AD brain tissue^5,13^.

In the present study, we investigated the magnetic properties of four post-mortem human brainstems using superconducting quantum interference device (SQUID) magnetometry. We focused on the brainstem since previous work revealed it to be the region with the highest magnetite concentrations throughout the brain^6^. We also examined iron-stained liver tissue from the corresponding subjects histologically since the liver plays a major role in iron metabolism^14^. Moreover, we employed laser ablation – inductively coupled plasma – mass spectrometry (LA-ICP-MS) to measure iron, copper and manganese in the brainstems, since a deficiency in hepatic metal removal in a dysfunctional liver can lead to the accumulation of trace metals in the brain^15^.

## Results

**Fig. 1:**
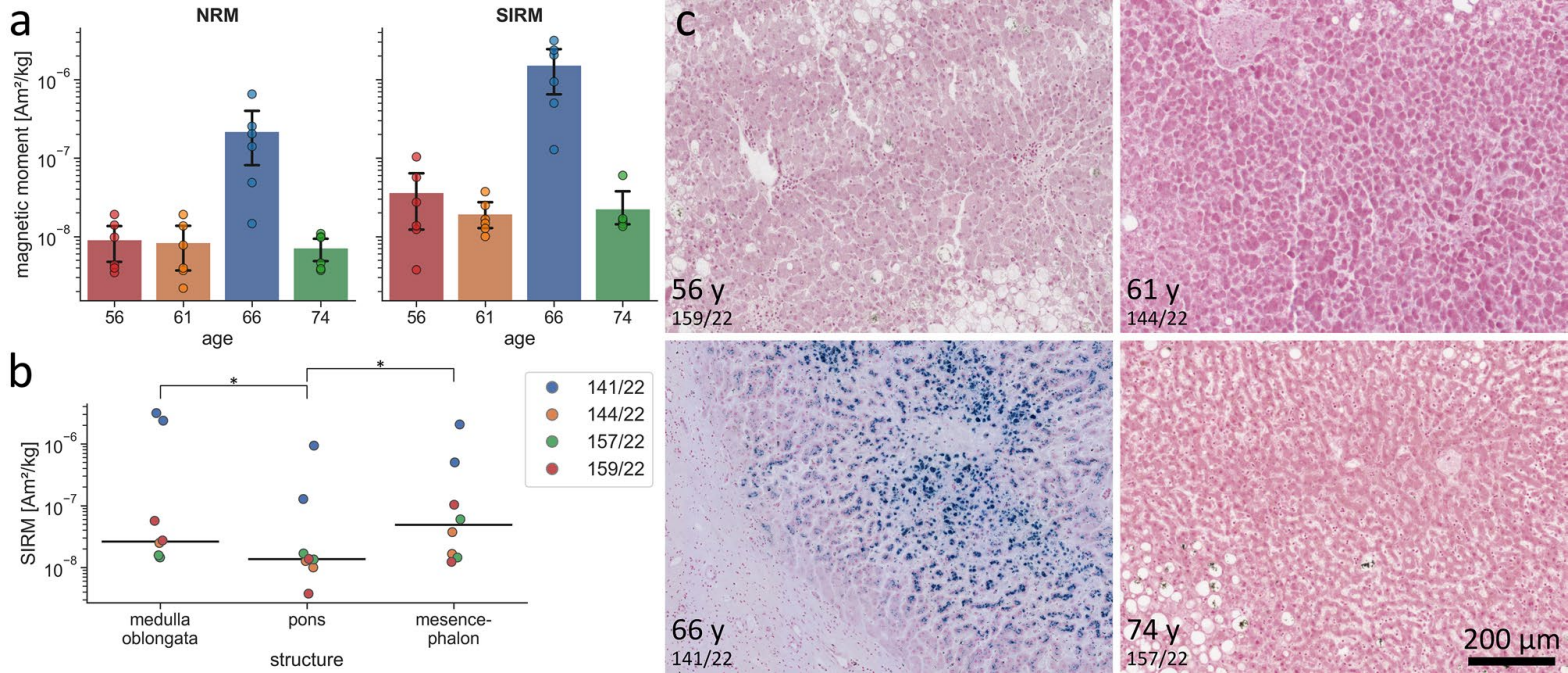
**Magnetometry results of brain tissue and pathohistological examination of liver tissue. a** Mass normalized natural remanent magnetization (NRM) and saturated isothermal remanent magnetization (SIRM) of the four investigated brainstems, ordered by age of the subjects (also see Supplementary Table 2). Data are represented as mean magnetic moments with 95% confidence intervals as well as individual data points. **b** Vertical distribution of mass normalized SIRM in the brainstems. Data are represented as median SIRM of each structure (black lines) and individual data points, *p < 0.05 according to two-sided Wilcoxon signed-rank tests. **c** Perl’s Prussian blue stained liver tissue from each case (also see Supplementary Fig. 2). Scale bar: 200 μm.

Brainstem samples from one case (141/22) had almost two orders of magnitude higher magnetic moments compared to three other cases (Fig. 1a and Supplementary Table 1). This was statistically significant for both natural remanent magnetizations (NRM) (*p* < 0.004) and saturated isothermal remanent magnetizations (SIRM) (*p* < 0.002) according to two-sided Mann-Whitney U tests. Most NRM, including that of case 141/22, and all SIRM exceeded the noise threshold of the magnetometer (Supplementary Fig. 1). The SIRM of the pons were significantly weaker than those of both the medulla oblongata (*p* = 0.023) and mesencephalon (*p* = 0.039) according to two-sided Wilcoxon signed-rank tests (Fig. 1b). Alternating field demagnetization curves revealed a coercivity distribution in left pons samples from cases 141/22 and 157/22 (Supplementary Fig. 1) that was consistent with the presence of magnetite in both cases.

Perl’s Prussian blue staining of liver sections revealed iron overload only in the liver of case 141/22 (Fig. 1c). Iron deposition in that liver followed a distinctive pattern (Supplementary Fig. 2) that led to the diagnosis of a secondary, non-hemochromatosis, iron overload disorder^16^ and is characteristic of alcohol-associated liver disease (ALD)^17^: a heterogeneous distribution from one lobule to another, lack of iron within fibrous septa, biliary cells and vascular walls, and iron loading of parenchymal cells and Kupffer cells. Further supporting the diagnosis of ALD for case 141/22 was the presence of severe liver cirrhosis, which was most likely caused by chronic alcohol abuse^18^. The livers of the three other cases showed no signs of cirrhosis or iron deposition, as can be expected from normal hepatic iron conditions^16^ (Fig. 1c).

**Fig. 2:**
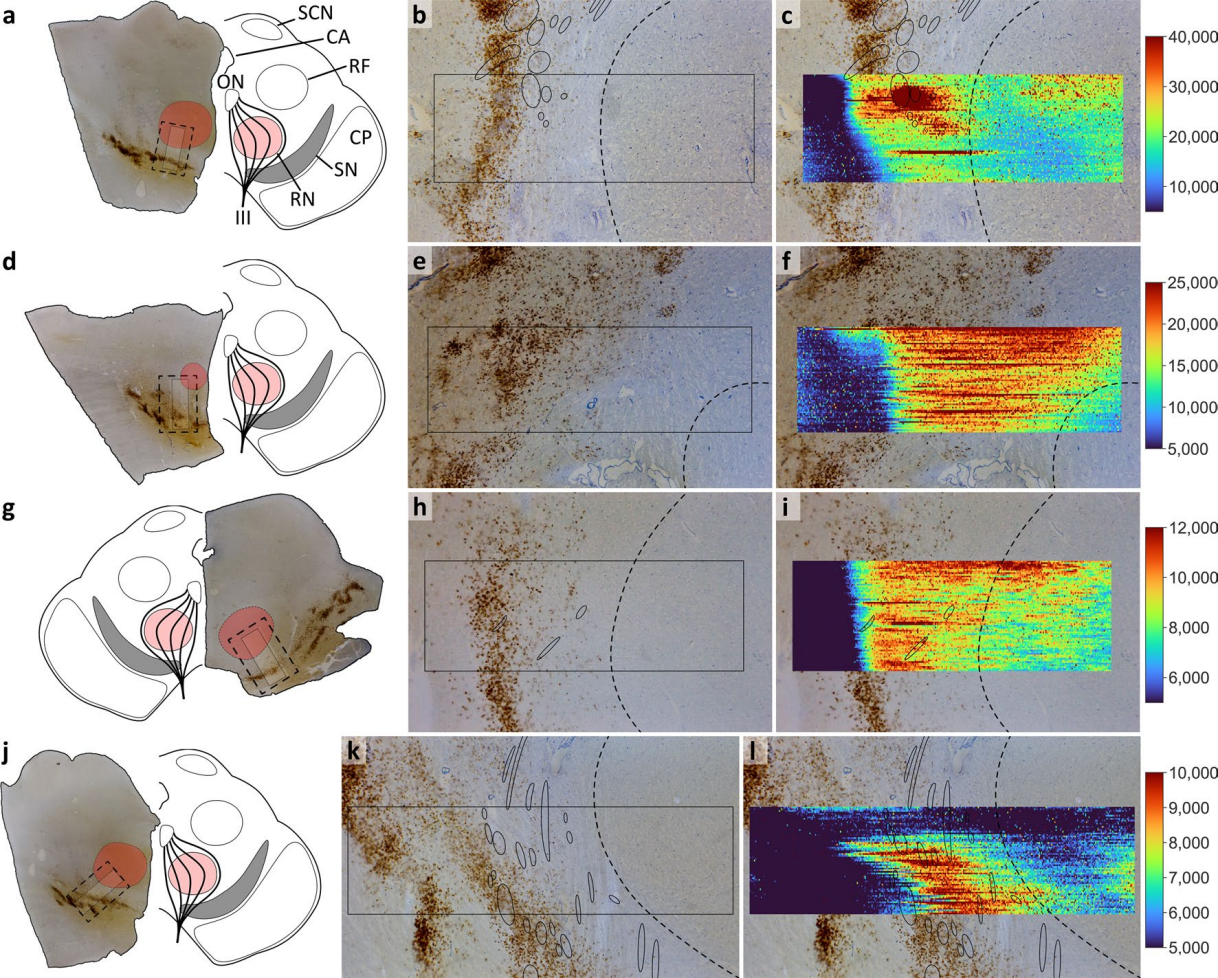
**Iron distribution measured by LA-ICP-MS in mesencephalon samples from each case. a-c** 141/22; **d-f** 144/22; **g-h** 157/22; **j-l** 159/22. **a**,**d**,**g**,**j** Images of the full sections and drawings illustrate the orientation of the samples as well as the fields of view (dashed rectangles) shown in the other panels. The overview of case 157/22 (**g**) was horizontally mirrored to show correct orientation. **b**,**e**,**h**,**k** Photomicrographs showing the structures scanned by LA-ICP-MS. Highly visible accumulations of neuromelanin-containing cells as part of the Substantia Nigra pars compacta (SNpc) were observed in each sample. The red nucleus (RN), identified as the oval region dorsomedial from the SNpc with higher cell densities and fewer nerve fibers than surrounding areas, is illustrated by a dashed line in all panels. Fibers of the oculomotor nerve are marked in continuous ovals. The sample for case 144/22 was cut more caudal, leading to no oculomotor fibers and causing the RN to appear smaller and with a larger distance to the SNpc. **c**,**f**,**i**,**l** ^56^Fe intensity distributions, with color scales adapted for each scan (arbitrary units). LA-ICP-MS scans (rectangles) covered an area of (**a-i**) 1.7 × 5 mm or (**j-l**) 1.7 × 6 mm. Abbreviations: SCN, superior colliculus nucleus; CA, cerebral aqueduct; ON, oculomotor nucleus; RF, reticular formation; CP, cerebral peduncle; III, third cranial (oculomotor) nerve.

We further measured iron distributions in brain sections from all four cases using LA-ICP-MS (Fig. 2). These distributions covered parts of the Substantia Nigra pars compacta (SNpc) and the Red Nucleus (RN). The highest iron intensities were observed in the white matter region in between SNpc and RN in all four cases. Iron measured with LA-ICP-MS (Fig. 2) represents the total iron detected in examined brain regions, including heme- and ferritin-bound iron, as well as other non-magnetic iron. Magnetic iron phases, as detected with magnetometry measurements (Fig. 1), only constitute a small fraction of the total iron^2^.

**Fig. 3:**
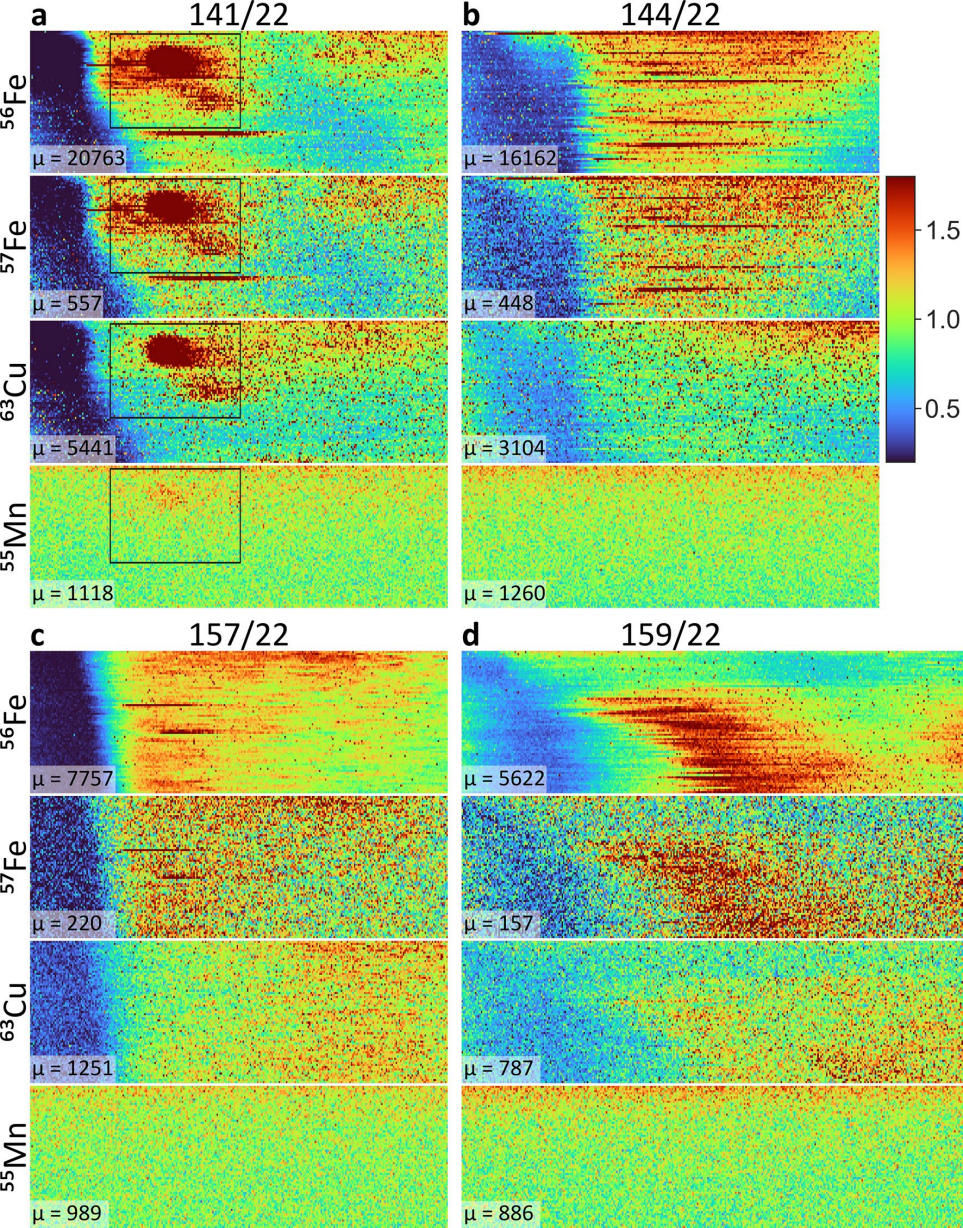
**Two-dimensional distributions of iron (**^**56**^**Fe and** ^**57**^**Fe)**,** copper (**^**63**^**Cu) and manganese (**^**55**^**Mn) measured by LA-ICP-MS for each case. a** 141/22; **b** 144/22; **c** 157/22; and **d** 159/22. Intensities were normalized by the mean intensity (µ) of each distribution (given in lower left corner). Black rectangles illustrate the scanned regions for case 141/22 shown at higher magnification in Fig. 4.

The two iron isotopes, as well as ^63^Cu, colocalized well in all four cases (Fig. 3). ^56^Fe had a consistently higher signal-to-noise ratio than ^57^Fe, as expected given that ^56^Fe and ^57^Fe make up 91.8% and 2.1% of the iron isotopic composition in body tissue^19^. Total iron and copper levels were highest in the ALD case (Fig. 3 and Supplementary Table 3). Therefore, the ALD case, with significantly higher SIRM values, also showed elevated levels of total iron and copper (illustrated in Supplementary Fig. 3 for both mean and maximum ^56^Fe intensities).

**Fig. 4:**
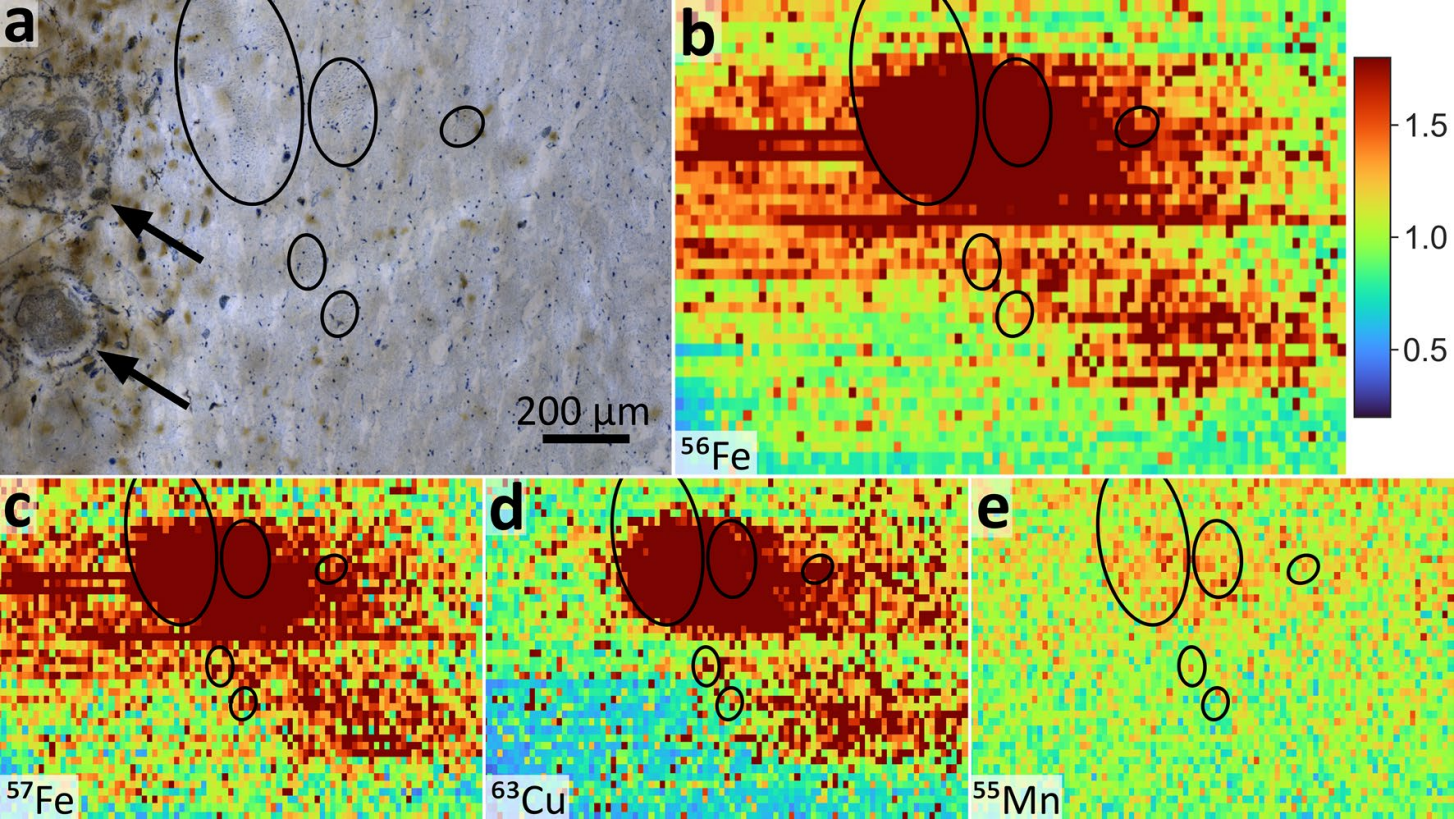
**High-magnification details of the LA-ICP-MS scans of case 141/22** (indicated by black rectangles in Fig. 3a). **a** Microphotograph of the Toluidin-blue stained section (Scale bar: 200 μm). **b-e** LA-ICP-MS data of (**b**) ^56^Fe, (**c**) ^57^Fe, (**d**) ^63^Cu and (**e**) ^55^Mn of the corresponding region. Intensities were normalized by the mean intensity of each scan (see also Fig. 3). Myelinated fibers from the oculomotor nerve passed through this region (black ovals). Two round polymerization artefacts (black arrows) did not coincide with the isotope distributions.

Parts of the oculomotor nerve identified as highly myelinated nerve fibers passing through the white matter between SNpc and RN were found in some of the investigated samples. For case 141/22, the oculomotor nerve fibers coincided with the main intensity peaks for all measured isotopes (Fig. 4). Given the relatively high scan speeds during LA-ICP-MS (100 μm/s, see Supplementary Table 4), the strong signal over the oculomotor fibers extended over the outline of the fibers most likely due to blurring and carry-over effects that are known for LA-ICP-MS^20^. None of the other cases showed such an agreement between isotope signals and location of oculomotor nerve fibers (Fig. 2). Two round structures with uneven surfaces were identified as polymerization artefacts most likely caused by local excessive temperatures during polymerization (black arrows in Fig. 4). The upper artefact partly coincided with iron signals, but not with copper or manganese signals. The lower artefact did not show an overlap with any isotope signal. The artefacts therefore did not cause the isotope signals, and the maximum seen in the intensity distributions of all four isotopes can be attributed to oculomotor nerve fibers. ^55^Mn had the highest noise levels of all four isotopes. For case 141/22, a peak in ^55^Mn was observed in the oculomotor fibers (Fig. 4). Given that this ^55^Mn signal colocalizes with ^56^Fe, ^57^Fe and ^63^Cu, it is considered a robust signal. No comparable manganese signals were found in the other three cases.

## Discussion

Magnetite has ubiquitously been found as the carrier of magnetic remanence in human brain tissue^5–13^. SIRM can be used to estimate the concentrations of magnetite particles in brain tissue, as has been demonstrated by a combination of magnetic measurements with transmission electron microscope imaging, electron energy loss spectroscopy and energy-dispersive X-ray spectroscopy^7^. The magnetic moments of brain tissue investigated in this study, except for the ALD case 141/22, were similar to those reported in our previous work, where we found IRM acquisition curves of brain tissue samples to saturate by 200–300 mT, as is characteristic of magnetite^6^. The ALD and non-ALD cases had similar coercivity distributions consistent with a low-coercive, iron oxide phase (Supplementary Fig. 1), revealing that the magnetic moments from both originated from magnetite particles. Our measurements could not determine whether particles were present as magnetite or its oxidized form, maghemite. However, others have found magnetite nanoparticles with only little surface oxidation to be the dominant carriers of magnetic remanence in brain tissue samples^7^. The size of magnetite particles in tissue defines their magnetic properties. As we detected magnetic remanence of brain tissue samples at room temperature, we can conclude that the magnetite particles were larger than the superparamagnetic size threshold for magnetite of 25–30 nm at 300 K^21^. Others using electron microscopy have found that most magnetite particles in brain tissue are < 50–70 nm in size^7,8^with some larger particles measuring up to 150 nm^7^ or even 600 nm^8^. We therefore conclude that the magnetic remanence carriers in our samples were magnetite nanoparticles.

The SIRM values of brain tissue recently reported by others^5^ were about one order of magnitude larger than the SIRM values of the non-ALD cases determined here. However, mass normalizations in Hammond et al.^5^ were based on the weights of freeze-dried tissue, whereas SIRM values in our present study and in Gilder et al.^6^ were normalized by wet weight. Freeze-drying decreases the weight of formalin-fixed brain tissue by approximately a factor of six^13, ^explaining the observed differences.

Magnetite nanoparticles have been detected within the core of amyloid-β plaques, leading to the hypothesis that they are associated with AD^11,12^. However, none of the four cases of the present study, including 141/22, showed signs of neuropathological conditions, thus excluding amyloid-β plaques as a potential explanation for the high concentrations of magnetite in 141/22. Magnetite in the brain has also been hypothesized to originate from exogenous, pollution-based particles^5,7,10^. However, individuals from Mexico City (Mexico) with extremely high air-pollution levels were reported with only five times higher magnetite concentrations in the brain compared to individuals from Manchester (UK)^5,10^. It is therefore unlikely that the observed differences in magnetite concentrations were caused by a difference in air-pollution exposure, particularly given that the samples were collected in Germany, where air-pollution levels are generally lower compared to Mexico City.

Instead, we found case 141/22 to show massive hepatic iron overload and a severely damaged liver, which is characteristic of ALD (Fig. 1c). Liver failure can result in elevated systemic iron levels through a number of mechanisms, including decreased hepcidin expression, elevated transferrin and ferritin saturation, and an inability to remove excess iron from the labile iron pool^14^. Furthermore, liver damage can cause increased levels of iron, copper and manganese in the brain^23–27^. These trace metals influence the cellular redox balance and participate in the formation of reactive oxygen species^27^ and can subsequently lead to hepatic encephalopathy^15^. We found increased levels of these trace metals in the ALD brainstem, which aligns with the described mechanisms for liver damage^23–27^. For ALD in particular, increased iron concentration in the brain is a known pathogenesis^25^. In addition, chronic alcohol abuse can lead to a disruption of the blood-brain barrier^28^.

The LA-ICP-MS data further showed systematic distributions with maximum intensities in the white matter in between the SNpc and RN. Iron in the brain is generally more abundant in white matter than in gray matter^30^. Even in nuclei that are typically identified as iron-enriched, such as the basal ganglia, the cerebellar nuclei, and the SN, the cells with the highest iron levels are the myelin-forming oligodendrocytes^30,31^. Regarding the SN, iron in healthy individuals is mainly located in the Substantia Nigra reticulata (SNr), which is extensively myelinated by large amounts of oligodendrocytes, whereas the SNpc is not enriched in iron in healthy individuals^30^. This has recently been confirmed with LA-ICP-MS in rodent brains^32^. Given that the SNr is ventrally located relative to the SNpc, the LA-ICP-MS scans in this study did not cover the iron-enriched SNr. The investigated tissue samples also showed no signs of Parkinson’s disease, for which substantially increased iron in neuromelanin-rich regions of the SNpc would be a typical finding^1^. Copper in the brain is also typically stored in glial cells and predominantly found in the locus coeruleus and the SN^33^. It also linearly correlates with iron under physiological conditions^34^, as confirmed by our data (Fig. 3). Moreover, the only valid manganese signal arose in the ALD case. Increased manganese concentrations in the SN are a typical finding in hepatic encephalopathy^23^. We detected manganese in the oculomotor nerve fibers that passed next to the SN (Fig. 4). These fibers also showed the highest iron and copper signals. Fibers from the oculomotor nerve showed excessive iron deposition in cases of progressive supranuclear palsy^35^. No signs of progressive supranuclear palsy were present for case 141/22, and whether ALD leads to a specific accumulation of iron in the oculomotor nerve is unknown. However, symptoms of chronic alcohol abuse include oculomotor signs as part of Wernicke’s syndrome^36^. The oculomotor nerve is also heavily myelinated, and myelin-formation in oligodendrocytes requires high amounts of iron^31^.

The ALD case of the present study showed typical hepatic iron overload and elevated levels of iron, copper and manganese in the brain, which was not unexpected given that trace metals and in particular iron can accumulate in the liver and the brain of ALD patients. We also found exceedingly high concentrations of magnetite nanoparticles in the brain tissue of the ALD case. Therefore, we suggest that a damaged liver with concurrent hepatic iron overload, such as in ALD, can cause magnetite nanoparticles to accumulate in the brain. Considering these findings, a distribution towards the brainstem and cerebellum^6^ may be attributed to the blood supply to the brain since the vertebrobasilar system, which supplies the posterior brain with oxygen-rich blood, runs directly alongside the brainstem. Our hypothesis is also supported by the repeated findings of high concentrations of magnetite in the meninges^8,37^. Based on the results of the present study, the origin of magnetite particles in the body remains unknown, since our measurements cannot be used to differentiate between exogenous and endogenous particles, which could both be transported to the brain in blood. Exogenous particles have previously been suggested to reach the brain through the olfactory bulb^5,7^ or the neuroenteric system and the vagal nerve^10^. However, the olfactory nerve enters the brain in the frontal lobe, where relatively low concentrations of magnetite were found^6^. On the other hand, pollution-based magnetite particles may get into the blood stream by a take-up through enterocytes or penetration through the respiratory epithelial barrier. Similarly, endogenous magnetite particles may be formed in organs other than the brain, such as the liver, which has been shown to have elevated magnetite concentrations^37^.

Collectively, the findings of this study indicate that magnetite particles can accumulate in the human brain as a consequence of liver damage. This mechanism has not been considered for magnetite occurrence in the brain so far. Several authors reported large variations in magnetite concentrations in brain tissue, with speculations about the underlying reasons ranging from hypothetically different levels of air-pollution exposure to schizophrenia^5,6^. However, potential concomitant liver pathology has not yet been considered in any study on magnetite in the brain, and thus, these hypotheses^5,6^ should be interpreted cautiously. The results presented here clearly indicate that future research on magnetite in the brain should consider liver pathology.

## Materials and methods

### Brain tissue samples

Post-mortem brainstems from four subjects were collected at the Institute of Forensic Medicine at the University of Rostock (Germany). A forensic autopsy was performed as requested by the public prosecutor and approved by a judicial decision. For this type of autopsy, formal consent is not required. Furthermore, the study was approved by the Local Ethical Committee at the Medical University of Rostock (ethical approval number: A 2021 − 0282). All work was conducted in accordance with official regulations and guidelines by the state of Mecklenburg-Vorpommern, Germany. Age, sex, the cause of death and the post-mortem interval were known for each individual, which otherwise remained anonymous (Supplementary Table 2). The individuals died for reasons unrelated to the brain, and the four brains showed no signs of neurological damage. Extraction followed standard procedures, with much attention paid to minimize the risk of contaminating the tissue with magnetic particles. Solely the opening of the skull was done with a metallic saw, but care was taken not to damage the dura mater. During the skull opening, the brainstem was protected from potential magnetic contaminants by the rest of the overlying brain. For the following extraction and dissections, only ceramic tools that had been washed with 10% HCl and filtered distilled water were used. Immediately after extraction, the brainstems were stored in sterile plastic bags at −20 °C.

The four brainstems were immersion fixed in buffered, filtered 10% formaldehyde for five days. During fixation, daily measurements of the pH-value were taken to monitor formaldehyde acidity. The formaldehyde was replaced twice during fixation to ensure a stable pH-value of 7.0. This procedure should not have altered the magnetic properties of the tissue^13^. Even if fixation lowered magnetite concentrations, magnetic moments would be systematically lowered in all brainstems since they were treated identically. After fixation, the pia and arachnoid mater as well as large blood vessels were removed. Each brainstem was divided by two horizontal cuts (separating the medulla oblongata, pons and mesencephalon) and one sagittal cut (separating the left and right portions of each structure), resulting in six tissue samples per brainstem. Tissue samples were weighed and stored in sterile plastic cups, which were washed with 10% HCl and filtered distilled water. Autopsy, fixation, cutting and storage protocols of all four brainstems were identical and performed on the same day except for the autopsy, which took place in the preceding weeks.

Liver tissue samples from biopsies were embedded in paraffin following standard protocols. For each case, one 5 μm-thin paraffin section was stained with hematoxylin-eosin (HE) and a second one with Perl’s Prussian blue.

### Magnetic moment measurements

Magnetic moments of the tissue samples were measured following existing protocols^6^. In short, the full vector magnetic moment of each sample was measured with a three-axis superconducting magnetometer (2G Enterprises Inc., Mountain View, CA, USA) at room temperature. Tissue samples were first measured in their unmagnetized, natural state (natural remanent magnetization, NRM). They were then exposed to a 0.63 T magnetic field using an electromagnet, followed by measurement of their acquired saturated isothermal remanent magnetization (SIRM). Each measurement consisted of four recordings, with the tissue sample being rotated 90° four times in the x-y plane. The four recordings were averaged, and baseline measurements (without tissue sample) subtracted. The magnetic moment measurements were repeated for 22 different samples in order to define the instrument noise level. Magnetic moments were recorded using CryoMag software^38^.

The magnetometer was situated in a magnetically shielded room (approximately 200 nT ambient field) in a forest 80 km northeast of Munich (Germany). In addition to fewer anthropogenic magnetic particles in the air of a forest compared to urban environments^39^, the magnetically shielded room was converted to a clean room before measurements to minimize the risk of magnetic contamination. Concerted effort was taken to perform the measurements in a magnetically clean environment: (i) inflowing air was filtered (high efficiency particulate air [HEPA] filter and electromagnetic filter); (ii) only one person handled samples and the sample holder while a second person operated the measuring system; (iii) both persons wore personal clean room equipment; and (iv) the exposure of samples to air was kept to a minimum (about two minutes for each measurement).

### Trace metal analyses

Distributions of iron, copper and manganese in the brain tissue were measured using LA-ICP-MS. Data were recorded on one mesencephalon sample from each case (157/22, left hemisphere; other cases, right hemisphere). LA-ICP-MS measured the two-dimensional distribution of selected isotopes by ablating particles from the sample surface with a laser, which were directed to the MS by Argon gas. To achieve planar sample surfaces, mesencephalon samples from each of the four brainstems were embedded in methylmethacrylate (MMA) according to existing protocols^40,41^. To this end, the samples were first dehydrated in ethanol baths of increasing concentration (70, 80, 90, 100%), then degreased in xylene, followed by incubation in 100% methanol. Finally, the samples were embedded in MMA (product number: 800590, Sigma Aldrich, St. Louis, MO, USA). To minimize contamination with magnetic particles, chemicals were filtered with PTFE filters (mesh size, 0.05 μm) except for more viscous chemicals that were filtered with a 0.2 μm mesh. The chemicals were filtered into glasses that were pre-washed with 10% HCl and rinsed with filtered distilled water. Slices of approximately 600 μm in thickness were cut from the MMA blocks using a circular saw microtome (SP 1600, Leica, Wetzlar, Germany). The slices were ground and polished with a 400 CS micro-grinder (EXAKT Advanced Technologies, Norderstedt, Germany), which decreased the slice thicknesses to approximately 400 μm; surfaces were thoroughly cleaned with isopropanol after polishing.

The laser ablation system (NWR-213, New Wave Research Inc., Fremont, CA, USA) couples to a NexION 300 ICP-MS (PerkinElmer, Waltham, MA, USA), with continuous recordings of iron isotopes (^56^Fe, ^57^Fe), copper (^63^Cu) and manganese (^55^Mn) (Supplementary Table 4). For each sample, a 5 × 1.7 mm area was selected covering parts of the Substantia nigra pars compacta (SNpc), which was easily visible due to large amounts of cells containing neuromelanin and is in general an area of interest for metals in the brain. Neuromelanin in the SNpc was used for orientation since the samples remained unstained for LA-ICP-MS to avoid possible contamination. The scanned area was extended from the SNpc to the red nucleus (RN) when possible. For case 159/22, the area was increased to 6 × 1.7 mm to scan a larger portion of the RN. The scans were oriented in a similar way, with the SNpc on the left side of the scanned area (i.e., during each line, the laser ablated first from the SNpc, then from white matter and lastly from the RN). Scans were done line-wise with 67 lines per scan; intensities were uncalibrated. For each scan, data from the first ten seconds of each line were discarded due to the delay between laser ablation and ICP-MS measurement; data were analyzed using Laser Ablation App 1.0 (unpublished).

### Light microscope analyses

Following LA-ICP-MS, the brainstem sections were stained with Toluidine blue and imaged using a digital single-lens reflex camera (EOS 5D, Canon Inc., Ōta, Tokyo, Japan) equipped with a macro objective (LM macroscope 42x XL, Micro Tech Lab, Graz, Austria). Stained liver tissue samples were imaged with a light microscope (Axio imager.M2, ZEISS, Oberkochen, Germany) equipped with a 40x objective (Plan-Apochromat 40x, ZEISS, Germany) and operated with Stereo Investigator software (MBF Bioscience, Williston, VT, USA). Images were analyzed using an image browser (Biolucida Viewer, MBF Bioscience). Figures were constructed using a raster graphics editor (Affinity Photo 2, Serif Europe Ltd., Nottingham, UK). Only contrast and brightness adjustments were made without altering the appearance of the original materials.

#### Statistical analysis

Data were analyzed with customized scripts in Python programming language (version 3.9, Python Software Foundation, Wilmington, DE, USA). Statistical tests included Mann-Whitney-U tests for independent samples and Wilcoxon signed-rank tests for dependent samples, both using confidence levels of 95%.

## Electronic supplementary material

Below is the link to the electronic supplementary material.

Supplementary Material 1

## Data Availability

All source data are available on the figshare repository at https://doi.org/10.6084/m9.figshare.25908568 (ref.42).
