# Supplementary material for: Magnetic iron-oxide nanoparticles in the brain connected to alcohol-associated liver disease
Source: Sci Rep. 2025 Jul 8;15:24505. doi: 10.1038/s41598-025-09756-8 (PMC12238357; doi:10.1038/s41598-025-09756-8)
Supplement: Supplementary file 1 — Supplementary Material 1 [file 41598_2025_9756_MOESM1_ESM.pdf]

Supplementary Information

Magnetic iron-oxide nanoparticles in the brain connected to alcohol-associated liver disease

Leon Kaub[1,2,*], Stefan Milz[2], Nirav Barapatre[2], Andreas Büttner[3], Bernhard Michalke[4], Christoph Schmitz[2], Stuart A. Gilder[1]

[1] Department of Earth and Environmental Science, LMU Munich, Munich, Germany
[2] Department of Anatomy II, Faculty of Medicine, LMU Munich, Munich, Germany
[3] Institute of Forensic Medicine, Rostock University Medical Center, Rostock, Germany
[4] Research Unit Analytical BioGeoChemistry, Helmholtz Center Munich, Neuherberg, Germany

*Corresponding author: Leon Kaub (leon.kaub@lmu.de)

Table of contents

Supplementary Figure 1	2
Supplementary Figure 2	3
Supplementary Figure 3	4
Supplementary Table 1	5
Supplementary Table 2	6
Supplementary Table 3	7
Supplementary Table 4	8

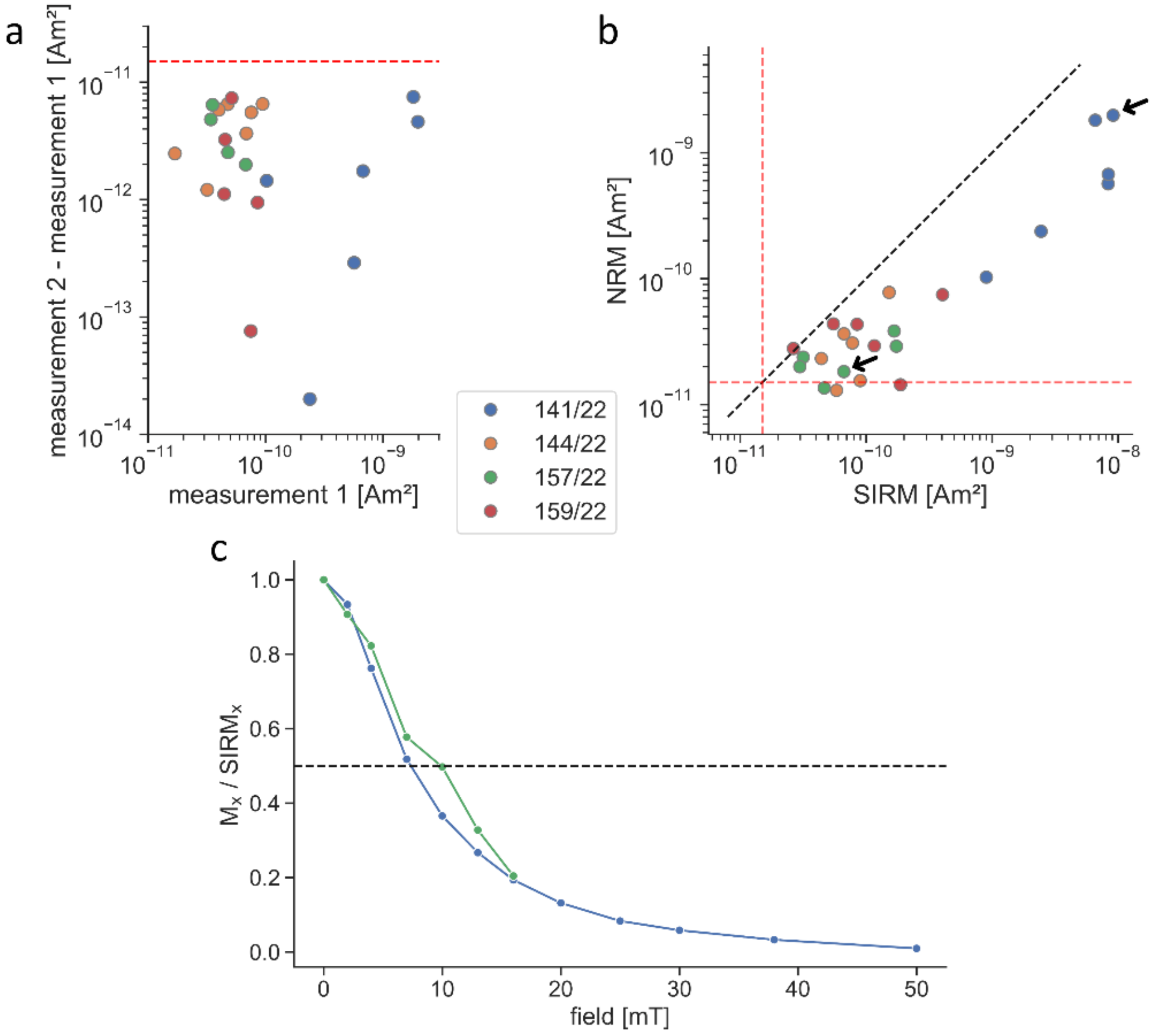

Supplementary Fig. 1. Magnetic measurements. a Differences of 22 repeat measurements (NRM and SIRM, not mass normalized), from which a conservative estimate for the instrument noise level was determined ($1.5x10^{-11}$ Am², red dashed line). **b** Non-mass normalized NRM values versus the corresponding, non-mass normalized SIRM values for all samples. All SIRM measurements were above the instrument noise level (red dashed lines); none of the four NRM values below the noise level come from case 141/22. **c** Stepwise alternating field (AF) demagnetization of left pons samples from 141/22 (blue) and 157/22 (green); samples indicated with black arrows in panel b). As the magnetizing field of the SIRM was along the samples' X-axes, AF demagnetization was likewise performed on the samples' X-axes. Both samples show similar coercivity distributions, with a median destructive field (field at which the SIRM decreases to 0.5 of its initial value, dashed line) of 7.6 mT (141/22) and 10.4 mT (157/22). Noisier spectrum for 157/22 is due to the two orders of magnitude weaker SIRM than for 141/22.

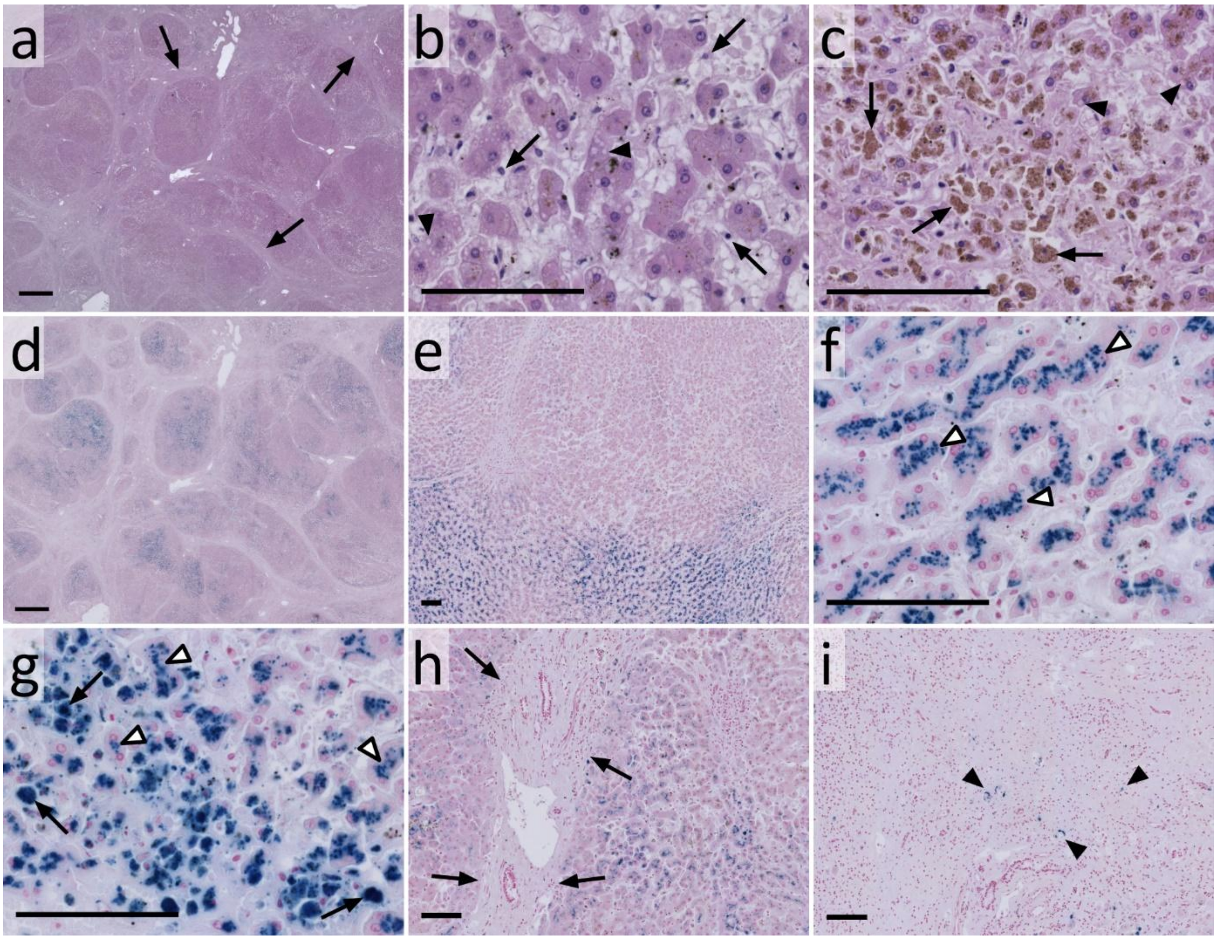

Supplementary Fig. 2. Representative photomicrographs of liver tissue from case 141/22 with (a-c) hematoxylin-eosin staining and with (d-i) Perl's Prussian blue staining that confirm the diagnosis of alcohol induced liver cirrhosis. a Severe liver cirrhosis can be seen by large fibrotic bands (arrows) that impacted the vascular architecture of the liver. **b** Large portions of the sinusoids were filled with fibrous tissue and cells (arrows), including fibrocytes and lymphocytes; hepatocytes in most places lost their plate-like structure. **c** Large amounts of lipofuscin and hemosiderin granules (arrows) were present in hepatocytes; lipid droplets were observed in parenchymal cells as intracellular, white globules (arrowheads in b and c). **d** Heavy iron overload revealed by Perl's Prussian blue staining, which turns iron-rich deposits blue. **e** Iron distribution was heterogeneous from one lobule to another. **f** Iron deposits were mainly found as granules at the biliary pole of the hepatocytes (arrowheads). **g** Massive iron accumulations were observed in Kupffer-cells and some hepatocytes, where individual granules were no longer resolvable (arrows). In contrast, easily distinguishable iron granules are seen in other hepatocytes (arrowheads). **h** Most bile ducts, endothelial cells and fibrotic tissue showed no iron deposits (arrows). **i** Some mesenchymal cells (endothelial cells and fibrocytes) within the large fibrotic bands were iron-positive (arrowheads). Scale bars: (a, d) 1 mm; (b, c, e-I) 100 µm.

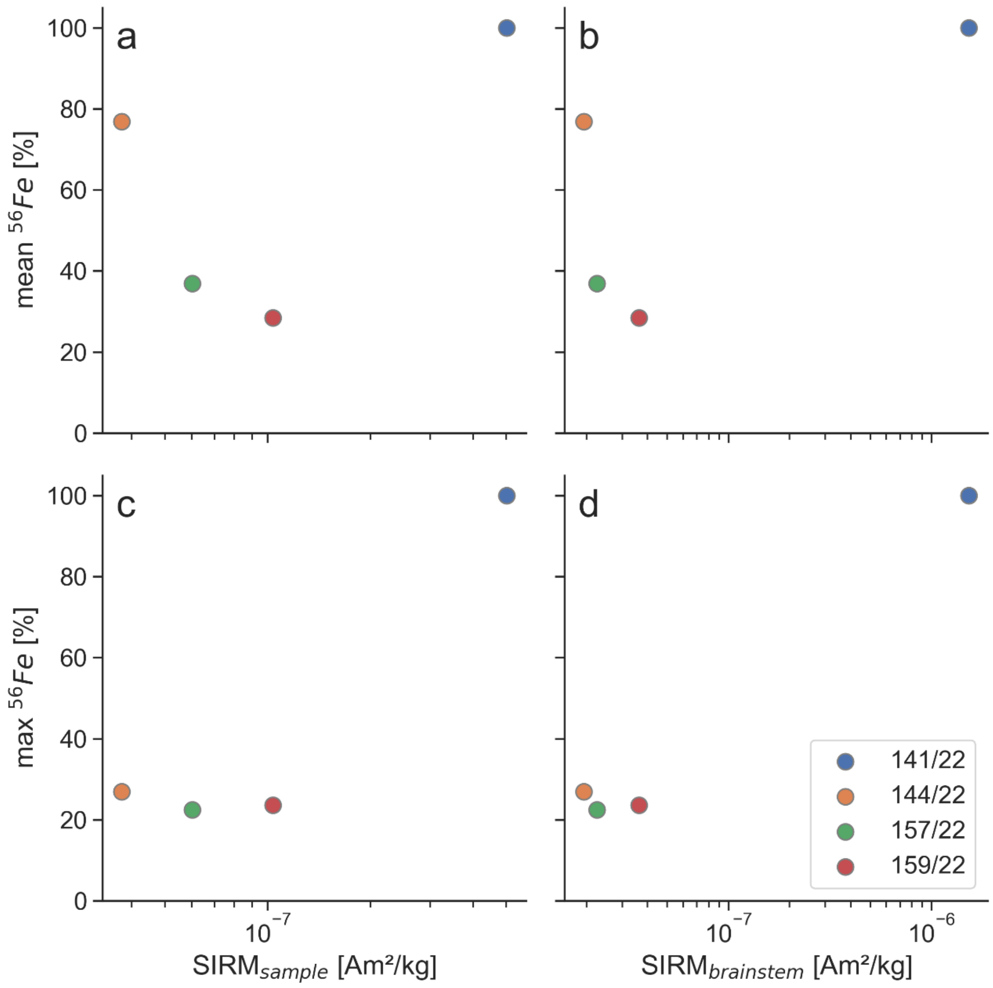

Supplementary Fig. 3. Mean and maximum ^{56}Fe intensities as a function of magnetic moment. LA-ICP-MS measurements were acquired from one brainstem samples for each case. Mean iron intensities are shown in (a) versus the SIRM of the corresponding sample that was chosen for LA-ICP-MS ($SIRM_{sample}$) and (b) versus the average magnetic moment of the corresponding brainstem ($SIRM_{brainstem}$) from the six samples in each case (Supplementary Table 1). Similarly, the maximum iron intensities are shown in (c) versus $SIRM_{sample}$ and (d) versus $SIRM_{brainstem}$. ^{56}Fe intensities were uncalibrated and are therefore illustrated in relative units with the mean and maximum intensity from case 141/22 as reference (resulting in values of 100% for case 141/22 in each panel). This direct comparison of isotope intensities between the samples is possible since all four samples were scanned with identical parameters on the same day (Supplementary Table 4). Supplementary Table 3 has uncalibrated statistical parameters of LA-ICP-MS measurements. The differences in mean iron intensities between case 141/22 and the three others were less than the differences in magnetic moments. LA-ICP-MS only measured small volumes (~ 0.1 mm³) of the samples that were measured in the magnetometer (~ 8 cm³). Since most of the ^{56}Fe intensities were found in the white matter, the overall differences between the four cases were also affected by the ratio of white to gray matter covered by the scan. Case 144/22 covered less of the RN and therefore more white matter (Fig. 2). The scanned areas of the three other cases had approximately similar ratios of white to gray matter. Therefore, the comparably high mean iron intensity of case 144/22 may have partly been caused by a larger ratio of white to gray matter.

Supplementary Table 1. Summary of magnetic moment data for the four brainstems.

Mag.	case	N	median [Am²/kg]	mean [Am²/kg]	sd [Am²/kg]	median / $median_{141/22}$	mean / $mean_{141/22}$
NRM	141/22	6	1.7E-07	2.2E-07	2.3E-07	1.000	1.000
	144/22	6	5.9E-09	8.4E-09	6.7E-09	0.035	0.038
	157/22	6	7.2E-09	7.2E-09	3.4E-09	0.042	0.033
	159/22	6	7.1E-09	9.1E-09	6.4E-09	0.042	0.042
SIRM	141/22	6	1.5E-06	1.5E-06	1.2E-06	1.000	1.000
	144/22	6	1.6E-08	1.9E-08	1.0E-08	0.011	0.013
	157/22	6	1.5E-08	2.3E-08	1.8E-08	0.010	0.015
	159/22	6	2.1E-08	3.6E-08	3.8E-08	0.014	0.024

Median and mean magnetization (Mag.) values and the standard deviation (sd) of the natural remanent magnetization (NRM) and the saturated isothermal remanent magnetization (SIRM) for each brainstem. The relative magnetic moments of each brain were normalized by case 141/22 for comparison. N, number of investigated samples.

Supplementary Table 2. Information about individuals from whom the brainstems originated.

case	age [yrs]	sex	post-mortem interval [hrs]	cause of death
141/22	66	Female	69	Alcohol induced liver cirrhosis
144/22	61	Male	45	Myocardial infarction
157/22	74	Female	9	Myocardial infarction
159/22	56	Male	41	Hemorrhagic shock

Supplementary Table 3. Statistics of LA-ICP-MS signals for each recorded isotope and sample.

Iso-tope	case	N	median	mean	sd	min	25th perc.	75th perc.	max
**^{56}Fe**	141/22	21760	18031	19671	16081	1180	12325	23620	515123
	144/22	21120	15118	15116	8924	2820	7742	19794	138751
	157/22	21440	8262	7265	3621	1060	4016	9503	115485
	159/22	24790	5341	5588	2346	1560	4041	6822	121434
**^{57}Fe**	141/22	21760	480	528	427	0	300	660	11184
	144/22	21120	400	423	242	20	240	560	3200
	157/22	21440	220	207	118	0	120	280	3921
	159/22	24790	140	156	87	0	100	200	5541
**^{63}Cu**	141/22	21760	4434	5170	4848	133	3034	5968	88272
	144/22	21120	2634	3158	2677	700	2100	3267	65282
	157/22	21440	1200	1188	1167	100	733	1467	63340
	159/22	24790	700	779	942	100	533	867	44402
**^{55}Mn**	141/22	21760	1100	1114	194	375	975	1225	3125
	144/22	21120	1250	1262	221	600	1100	1400	3450
	157/22	21440	975	989	171	300	875	1100	2725
	159/22	24790	875	887	173	350	775	1000	2300

Reported statistics include number of recordings (N), median, mean, standard deviation (sd), minimum (min), 25th percentile (25th perc.), 75th percentile (75th perc.), and maximum (max). Values are unitless due to uncalibrated LA-ICP-MS measurements.

Supplementary Table 4. Parameters for Laser Ablation - Inductively Coupled Plasma - Mass Spectrometry (LA-ICP-MS) measurements.

**LA**	Pulse repetition rate	10 Hz
	Spot size	25 µm
	Line spacing	25 µm
	Approx. ablation depth	10 µm
	Scan speed	100 µm/s
**ICP**	Plasma power	1300 W
	Nebulizer gas	0.98 L/min
	Plasma gas	15.8 L/min
	Auxiliary gas	1.2 L/min
**MS**	Registered isotopes	^{55}Mn, ^{56}Fe, ^{57}Fe, ^{63}Cu
	Dwell time/isotope	30 ms
